# Solvent Engineering Using a Volatile Solid for Highly Efficient and Stable Perovskite Solar Cells

**DOI:** 10.1002/advs.201903250

**Published:** 2020-03-10

**Authors:** Guohua Wu, Hua Li, Jian Cui, Yaohong Zhang, Selina Olthof, Shuai Chen, Zhike Liu, Dapeng Wang, Shengzhong (Frank) Liu

**Affiliations:** ^1^ Key Laboratory of Applied Surface and Colloid Chemistry National Ministry of Education Shaanxi Key Laboratory for Advanced Energy Devices Shaanxi Engineering Laboratory for Advanced Energy Technology School of Materials Science and Engineering Shaanxi Normal University Xi'an 710119 China; ^2^ Faculty of Informatics and Engineering The University of Electro‐Communications 1‐5‐1 Chofugaoka, Chofu Tokyo 182‐8585 Japan; ^3^ Department of Chemistry University of Cologne Luxemburger Street 116 50939 Cologne Germany

**Keywords:** crystal growth, perovskite solar cells, pinholes, solvent engineering, volatile

## Abstract

A strategy for efficaciously regulating perovskite crystallinity is proposed by using a volatile solid glycolic acid (HOCH_2_COOH, GA) in an FA_0.85_MA_0.15_PbI_3_ (FA: HC(NH_2_)_2_; MA: CH_3_NH_3_) perovskite precursor solution that is different from the common additive approach. Accompanied with the first dimethyl sulfoxide sublimation process, the subsequent sublimation of GA before 150 °C in the FA_0.85_MA_0.15_PbI_3_ perovskite film can artfully regulate the perovskite crystallinity without any residual after annealing. The improved film formation upon GA modification induced by the strong interaction between GA and Pb^2+^ delivers a champion power conversion efficiency (PCE) as high as 21.32%. In order to investigate the role of volatility in perovskite solar cells (PSCs), nonvolatile thioglycolic acid (HSCH_2_COOH, TGA) with a similar structure to GA is utilized as an additive reference. Large perovskite grains are obtained by TGA modification but with obvious pinholes, which directly leads to an increased defect density accompanied by a decline in PCE. Encouragingly, the champion PCE achieved for GA‐based PSC device (21.32%) is almost 13% or 20% higher than those of the control device or TGA‐based device. In addition, GA‐modified PSCs exhibit the best stability in light‐, thermal‐, and humidity‐based tests due to the improved film formation.

## Graphical abstract

A strategy is demonstrated for efficacious regulation of perovskite crystallinity using glycolic acid (GA) against nonvolatile thioglycolic acid (TGA) following dimethyl sulfoxide sublimation, resulting in enhanced device performance. A champion power conversion efficiency as high as 21.32% is achieved for the GA‐based device, which is almost 13% or 20% higher than those of the control device or TGA‐based device.

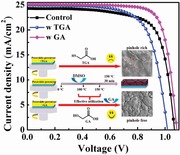

## 1 Introduction

Since Kojima et al. first reported organic–inorganic perovskite materials as visible‐light sensitizers in 2009,^1^ the power conversion efficiencies (PCEs) of hybrid perovskite solar cells (PSCs) have remarkably increased from the initial 3.8% to 25.2%^2^ in 2019. This demonstrates the potential of PSCs as an alternative to commercial silicon‐based solar cells for applications in the field of photovoltaics.^3^, ^4^, ^5^, ^6^, ^7^

Important for the performance of PSCs is a uniform and pinhole‐free surface morphology, good crystallinity, and large grain size throughout the perovskite film, all of which are strongly influenced by the nucleation and crystal growth process.^8^, ^9^, ^10^, ^11^, ^12^, ^13^ Organic–inorganic hybrid perovskite materials can be synthesized by the reaction of organic halides with Pb(II) halides.^14^, ^15^, ^16^ For the deposition, nucleation, and crystal growth process of perovskite films aprotic solvents such as *N*,*N*‐dimethylformamide (DMF),^17^ dimethyl sulfoxide (DMSO),^18^ dimethylacetamide (DMA),^19^ γ‐butyrolactone (GBL),^20^ hexamethylphosphoramide (HMPA),^21^ acetonitrile,^22^ 1,3‐dimethyl‐2‐imidazolidinone (DMI),^23^ 2‐methoxyethanol,^24^ *N*‐methyl‐2‐pyrrolidone^25^ and their mixtures like DMF/chlorobenzene/acetonitrile^26^ can be utilized. Furthermore, a wide range of additives like Pb(SCN)_2_,^27^, ^28^ PbI_2_,^29^ Pb(CH_3_COO)_2_,^30^ hydroquinone,^31^ methylammonium acetate,^32^ ammonium hypophosphite,^33^ trimethylammonium chloride,^34^ 2‐mercaptopyridine,^35^ etc. have been incorporated into the precursor solution to improve crystallinity and morphology or to reduce defect concentration in the perovskite film. Most of these reported solvent or additives possess a high boiling point of about or more than 200 °C and weak coordination abilities to PbI_2_. The latter effect would retard the crystallization rate of the organic–inorganic hybrid perovskite material due to the formation of an intermediate phase, thus promoting the growth of uniform films.^36^, ^37^

However, the residual of the unwanted phases is probably formed for the above common additive approaches. Another option to induce large grained perovskite formation with improved crystallographic orientation is to design perovskite precursor solutions with higher boiling points. Such processing will allow for higher tolerance regarding the delay time during the nucleation and crystal growth process of perovskite. The sublimation of DMSO from a perovskite film appears at less than 100 °C,^4^ so the key challenge is to design a weakly coordinating compound with a boiling point higher than DMSO but lower than the annealing temperature of the perovskite film such as FA_0.85_MA_0.15_PbI_3_ (FA: HC(NH_2_)_2_; MA: CH_3_NH_3_) (150 °C). It should only have a delaying impact on the nucleation and crystal growth behavior of the perovskite and vanish without a trace during the final annealing process. Such novel design strategy is required for the commercial large‐scale PSC production as it can substantially reduce the defect density of the perovskite films instead of the passivation methods like the common additive approaches.

In this work, we introduce the volatile molecule glycolic acid (HOCH_2_COOH, GA) which is solid at room temperature and has a moderate boiling point in an FA_0.85_MA_0.15_PbI_3_ perovskite precursor solution. GA as a polar molecule contains one carboxyl group in one side and one hydroxyl group in the other side. Thus, GA solid is facilely guaranteed to be dissolved in DMF/DMSO solvent. Compared with the other reported additives, one highlight of GA is the low boiling point less than 150 °C, there can be no residual of the unwanted phases expect perovskite after annealing. The interesting role of GA solid here plays more like a solvent rather than a common passivation additive. Before GA leaves the perovskite film, GA also can be utilized as a Lewis acid due to the relatively strong interaction between GA and perovskite including FA^+^ and PbI_2_. Due to this strong interaction, large perovskite grains can be obtained by GA modification. Finally, a champion PCE of 21.32% with outstanding stability was achieved for GA‐modified PSC due to the superior quality of the perovskite film that contained a lower number of defects. In order to investigate the effect of volatile versus nonvolatile molecules in perovskite solar cells, thioglycolic acid (HSCH_2_COOH, TGA) with a similar molecular structure to GA is utilized as an additive reference.

## 2 Result and Discussion

**Figure 1:**
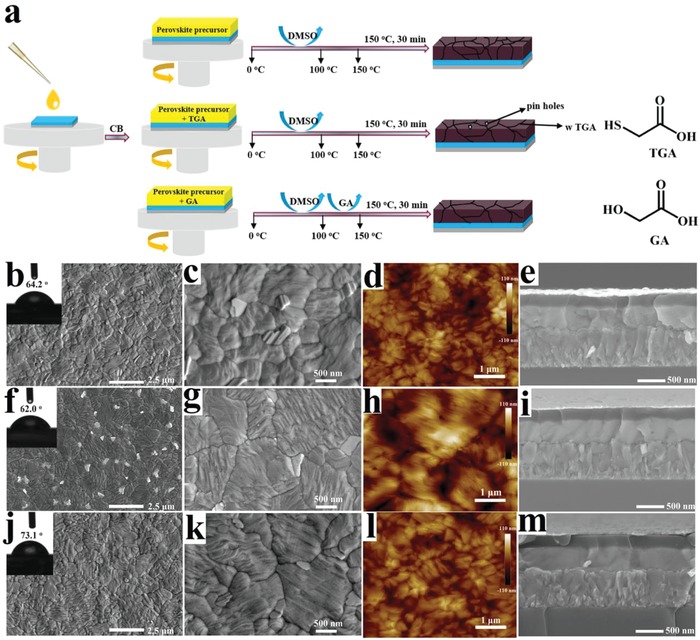
a) Schematic diagram of the preparation process of perovskite films with/without TGA or GA; molecular structures of TGA and GA are shown. The other figures show high‐resolution scanning electron microscopy (SEM), and atomic force microscopy (AFM) images of pristine (b–d), TGA (f–h), or GA (j–l) modified perovskite films. Inset: Contact angles of water droplets on the perovskite films. Further, cross‐section SEM images of pristine (e), TGA (i), or GA (m) modified PSC devices are shown with the structure FTO/TiO_2_/perovskite/spiro‐OMeTAD/Au.

As shown in **Figure** **1**a, the perovskite films were prepared via a typical process as described in our previous work (see Supporting Information for details).^38^, ^39^ In short, for the three compositions under investigation, either only a mixture of DMF and DMSO precursor solution was used, or GA or TGA was added into it. The precursors were then spin coated onto FTO/TiO_2_ substrates followed by an antisolvent drip of chlorobenzene (CB) solution. The film formation was completed by a 150 °C annealing step for 30 min. Figure 1 shows the scanning electron microscope (SEM) and atomic force microscope (AFM) images of pristine, GA‐, or TGA‐modified perovskite films. The corresponding water contact angle measurements are shown as insets. Upon the introduction of GA or TGA, the perovskite crystal grain size increases significantly, which is also obvious from the corresponding cross‐sectional SEM images of PSC devices in Figure 1. The interaction of perovskite with GA or TGA is likely to retard the perovskite crystal growth process during the early stage of the spin‐coating process, which facilitates the generation of these large grained perovskite films. From the AFM images we can extract the root‐mean‐square (RMS) roughness, which is 19.8 nm for the pristine perovskite film, 26.4 nm for TGA modification, and 17.2 nm for GA modification. Although the largest crystal grain sizes are observed for TGA modification, a rougher surface with pinholes is created. This is illustrated in more detail in Figure S1 (Supporting Information), where the concentrations of GA and TGA were furthermore varied. The pinhole formation is clearly unfavorable for the solar cell performance as it will produce shunting pathways in the device.^24^ In contrast, for the GA‐modified perovskite film, smooth and pinhole free surfaces are observed, beneficial for the enhancement of the PSC device performance.

**Figure 2:**
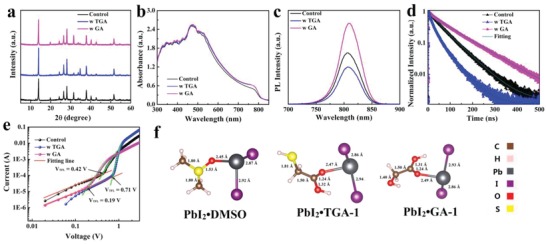
a) XRD patterns, b) absorption spectra, c) photoluminescence spectra, and d) transient‐state photoluminescence spectra of the perovskite films with and without GA or TGA. e) Dark current–voltage characteristics of electron only devices (FTO/TiO_2_/perovskite/PCBM/Ag) based on perovskite films with or without GA or TGA. f) Optimized molecular structures based on DFT calculations for PbI_2_⋅DMSO, PbI_2_⋅TGA‐1, and PbI_2_⋅GA‐1.

The effect of GA or TGA on the crystallinity of perovskite films is investigated by thin film X‐ray diffraction (XRD) and the measurement results are shown in **Figure** **2**a. In addition to the 3D perovskite related features, a diffraction signal at 12.5° originating from the (001) plane of the hexagonal PbI_2_ is observed for the pristine film^4^ and a very weak signal at 11.6° originating from the yellow phase of FA‐based perovskite (δ‐FAPbI_3_) is observed for TGA‐modified perovskite film.^40^ The addition of GA does not alter the crystal orientation relative to the pristine perovskite film, but the crystallinity is improved with the enhanced XRD intensity.^41^, ^42^, ^43^ In contrast, the addition of TGA affects the crystal orientation, resulting in an increased intensity of the (101) peak at 13.9° relative to the (211) peak at 31.4°, their ratio changes from 2.55 to 9.52.^44^ The ultraviolet–visible (UV–vis) absorption spectra of the perovskite films with and without GA or TGA are shown in Figure 2b. The absorption onset, i.e., band gap, of the perovskite films is not affected however the absorption of the perovskite film is slightly enhanced in the long wavelength region upon the GA or TGA modification. This should be beneficial in PSCs by enhancing the short‐circuit current (*J*_sc_). Next, steady‐state photoluminescence (PL) and time‐resolved photoluminescence (TRPL) decay measurements were performed to investigate the charge recombination dynamics in the different perovskite films as shown in Figure 2c,d. Compared with the pristine perovskite film, the PL intensity of the GA‐modified perovskite film is distinctly enhanced while the TGA‐modified perovskite film shows a weaker PL signal, likely due to the presence of pinholes. In agreement with the absorption measurement, no change in emission wavelength is found (807 nm) for these three perovskite films. The TRPL decay spectra can all be fitted by biexponential equations using a slower (τ_1_) and a faster (τ_2_) time constant.^17^, ^45^ The results are summarized in Table S1 (Supporting Information). The faster time is related to nonradiative recombination in the perovskite film induced by the charge trapping defect states. Meanwhile, the slower one originates from a direct recombination of free carriers.^46^ With the addition of GA, the fast decay lifetime increases from 25.43 to 28.61 ns and its percentage is reduced from 12.12% to 7.93% relative to the pristine film. Simultaneously, the slow decay lifetime is enhanced from 64.40 to 102.85 ns (percentage increases from 87.88% to 92.07%). In contrast, both the fast decay lifetime (from 25.43 to 16.35 ns) and the slow decay lifetime (from 64.40 to 48.64 ns) significantly decrease with TGA addition. This indicates a lower trap density in GA‐modified perovskite film and the higher one for TGA. To quantify the density of trap states in these perovskite films, space‐charge‐limited current (SCLC) measurements in the dark were performed as shown in Figure 2e and the sample structure of the electron only device was FTO/TiO_2_/perovskite/PCBM/Ag. The trap‐state density (*n*_trap_) can be calculated based on the equation *n*_trap_ = (2*εε*_0_ *V*_TFL_)/(*eL* ^2^),^47^, ^48^, ^49^, ^50^ in which ε and ε_0_ represent the relative dielectric constant of the perovskite film and the vacuum permittivity (8.854 × 10^−14^ F cm^−1^),^51^, ^52^ *V*_TFL_ can be extracted from the kink point between the ohmic and the trap‐filled limit region, e is the elementary charge, and *L* is the thickness of the perovskite layer obtained from the cross‐sectional SEM image. The extracted trap densities are 1.06  ×  10^16^, 1.80 ×  10^16^, and 0.48 ×  10^16^ cm^−3^ for the pristine, TGA‐modified, and GA‐modified perovskite films, respectively. The observed trend is consistent with the PL and TRPL results. We suggest that GA‐modified perovskite layers exhibit the smallest trap‐state density due to a reduction in Pb‐I antisite defects (induced by the undercoordinated Pb atoms), while the larger trap state density in TGA‐modified perovskite likely originates from the TGA residue and the numerous of pinholes.

To better understand the interaction between GA/TGA and the perovskite on a molecular scale, we performed theoretical calculations regarding their binding energy. The corresponding results are listed in Table S2 (Supporting Information). Due to the asymmetric structure of the modifiers, there are two possible conformations between GA/TGA and PbI_2_/FA^+^. The PbI_2_/FA^+^ can either interact with the oxygen on the carboxyl groups (referred to as “−1” below) or with the oxygen or sulfur on the hydroxyl or thiol group (called “−2”). The optimized molecular structures of PbI_2_⋅DMSO, PbI_2_⋅GA‐1 and PbI_2_⋅TGA‐1 are plotted in Figure 2f while the remaining ones for DMSO, TGA, GA, PbI_2_⋅DMSO, PbI_2_⋅GA‐2, and PbI_2_⋅TGA‐2 are shown in Figure S2 (Supporting Information). For the first case, the interaction energies of the bonds are calculated to be −0.955 eV for PbI_2_⋅GA‐1, and −0.989 eV for PbI_2_⋅TGA‐1. For the second case, these values are smaller (‐0.613 eV for PbI_2_⋅GA‐2, and −0.817 PbI_2_⋅TGA‐2). Thus, PbI_2_ prefers to react with the oxygen on the carboxyl groups in either GA or TGA. Further, the interaction energy between PbI_2_ and the oxygen in DMSO was calculated to be −0.840 eV. Overall, PbI_2_ can interact with either DMSO, GA, or TGA however a slightly stronger interaction of PbI_2_ with GA and TGA is found. To consider also the other components of the precursor solution, the binding energies of FA^+^⋅DMSO, FA^+^⋅ GA, and FA^+^⋅TGA were analyzed, yielding values of −1.347 eV (FA^+^⋅DMSO), −1.286 eV (FA^+^⋅GA‐1 or FA^+^⋅GA‐2), and −1.214 eV (FA^+^⋅TGA‐1) or −1.254 eV (FA^+^⋅TGA‐2). The optimized molecular structures of FA^+^⋅DMSO, FA^+^⋅TGA, and FA^+^⋅GA with the largest binding energy are shown in Figure S3 (Supporting Information). Intriguingly, this trend is different from the PbI_2_ adducts. FA^+^ preferentially interacts with DMSO, whereas PbI_2_ preferentially interacts with GA or TGA molecule. To probe such interactions between FA^+^ and GA/TGA experimentally, the three deuterated dimethylsulfoxide (DMSO) perovskite precursor solutions were probed by ^1^H NMR measurements as shown in Figures S4–S6 (Supporting Information). Upon the addition of GA or TGA, the ^1^H NMR signals of FA^+^ at 8.56 and 7.79 ppm split while the ^1^H NMR signals from the carboxyl groups in GA (12.37) or TGA (12.67) are in also displaced upfield, which strongly indicates the interaction of FA^+^ with GA or TGA.

**Figure 3:**
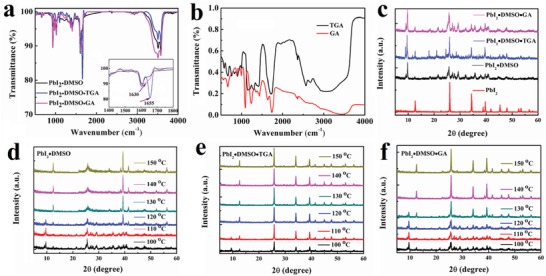
Fourier‐transform infrared (FT‐IR) spectra of PbI_2_ adducts (a) and GA/TGA molecules (b), XRD patterns of the as‐formed PbI_2_ adducts compared with PbI_2_ (c), annealed PbI_2_⋅DMSO adduct (d), annealed PbI_2_⋅DMSO⋅GA adduct (e), and annealed PbI_2_⋅DMSO⋅TGA adduct (f).

We further studied the influence of GA and TGA using Fourier transform infrared spectroscopy (FTIR). **Figure** **3**a,b shows such spectra for GA, TGA, PbI_2_⋅DMSO, PbI_2_⋅DMSO⋅GA, and PbI_2_⋅DMSO⋅TGA powder. The asymmetrical stretching vibrations related to the C=O bond in the pure GA and TGA, as shown in Figure 3b, appears at 1628 and 1623 cm^−1^, respectively. The vibrations is shifted to 1630 cm^−1^ for the mixture of PbI_2_⋅DMSO⋅GA and even to 1655 cm^−1^ for PbI_2_⋅DMSO⋅TGA. This indicates that TGA or GA molecules are successfully introduced into the corresponding powder. Next, Figure 3c–f shows the XRD patterns for as‐prepared and annealed PbI_2_, PbI_2_⋅DMSO, PbI_2_⋅DMSO⋅GA, and PbI_2_⋅DMSO⋅TGA films, respectively. For the as‐prepared films (Figure 3c), new XRD patterns compared to as purchased PbI_2_ appear if DMSO, DMSO/GA, or DMSO/TGA are included. This implies that PbI_2_ is transformed into a different compound upon the introduction of these molecules. As shown in Figure S7 (Supporting Information), there is a strong peak at 26.0° attributed to the (015) plane of PbI_2_,^53^ which is significantly changed to 25.7° for PbI_2_⋅DMSO, to 25.9° for PbI_2_⋅DMSO⋅GA, and it splits into two peaks at 25.6° and 25.9° for PbI_2_⋅DMSO⋅TGA. Upon annealing, the PbI_2_ lattice can be recovered via the release of DMSO and GA after a temperature of 150 °C for PbI_2_⋅DMSO⋅GA. The similar crystalline structure of PbI_2_ but with lower crystallinity was achieved for annealed PbI_2_⋅DMSO after a temperature of 150 °C compared with PbI_2_⋅DMSO⋅GA as shown in Figure S8 (Supporting Information), which demonstrated that GA modifier could improve the crystalline of PbI_2_ and would be beneficial to the perovskite crystalline. The annealing process is also supported by thermo gravimetric analysis (TG) on the PbI_2_⋅DMSO, PbI_2_⋅DMSO⋅GA, and PbI_2_⋅DMSO⋅TGA powders presented in Figure S9 (Supporting Information). Both PbI_2_⋅DMSO and PbI_2_⋅DMSO⋅GA show a single step loss at 127 and 136 °C, respectively. The PbI_2_⋅DMSO⋅GA complex exhibits a wider endotherm peak as the weight loss of both DMSO and GA appear during this process. PbI_2_⋅DMSO⋅TGA exhibits a more complicated weight loss possess with four different characteristic temperatures. The first three of these (91, 120, ≈168 °C) are attributed to the loss of DMSO while the fourth (≈220 °C) corresponds to the loss of TGA. This is a clear evidence that TGA remains in the film even after 150 °C annealing although annealed PbI_2_⋅DMSO⋅TGA exhibits the similar XRD patterns as annealed PbI_2_⋅DMSO⋅GA.

Also for the complete FAI perovskite adduct powders, these investigations were performed. The XRD patterns of as‐prepared FAI⋅PbI_2_⋅DMSO, FAI⋅PbI_2_⋅DMSO⋅GA, and FAI⋅PbI_2_⋅DMSO⋅TGA are shown in Figure S10a (Supporting Information). Again, distinct differences are found, showing the incorporation of the solvent and the modifiers in the perovskite film at room temperature. Exemplarily, the sample of FAI⋅PbI_2_⋅DMSO⋅GA is stepwise annealed as shown in Figure S10b (Supporting Information). Here, FAPbI_3_ is successfully formed after a heating at 150 °C for 30 min. The TG analysis on the as‐prepared powders of FAI⋅PbI_2_⋅DMSO, FAI⋅PbI_2_⋅DMSO⋅GA, and FAI⋅PbI_2_⋅DMSO⋅TGA are shown in Figure S10d–f (Supporting Information). Here, the weight loss of pure GA is at about 100 °C as shown in Figure S10c (Supporting Information) while for the pristine FAI⋅PbI_2_⋅DMSO perovskite powder (Figure S10d, Supporting Information) there are three main weight loss processes at approximately 90, 300, and 480 °C. These correspond to the loss of DMSO, FAI, and PbI_2_, respectively. For the GA or TGA incorporated perovskite powders, the loss temperature of FAI and PbI_2_ are higher than that of the pure FAI⋅PbI_2_⋅DMSO mixture, which demonstrates the strong interaction between the GA or TGA and perovskite precursor. There are two endotherm peaks at 86 °C (the weight loss of DMSO) and 212 °C (the weight loss of TGA) as shown in Figure S10e (Supporting Information) and two endotherm peaks at 92 °C (the weight loss of DMSO) and 139 °C (the weight loss of GA) as shown in Figure S10f (Supporting Information), which confirms of our previous findings that GA can disappear before 150 °C, while TGA still exists in the as‐annealed powder due to its high boiling point over 150 °C. This presence of TGA in the annealed film was further proven by energy‐dispersive X‐ray spectrometry (EDX). As the sulfur is specific to this modifier, one can analyze the film composition as shown in Figure S11 (Supporting Information). By comparing two different areas on the sample, we can conclude that TGA remains at the perovskite grain boundaries after such a moderate annealing step.

**Figure 4:**
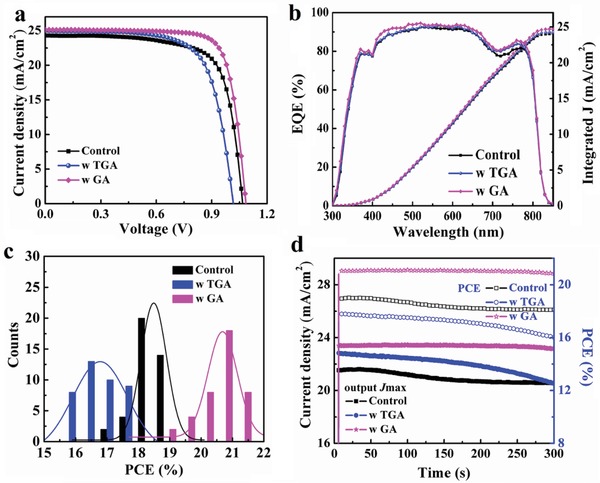
a) *J−V* curves under 1 Sun (AM 1.5G, 100 mW cm^−2^), b) incident photon‐to‐electron conversion efficiency (IPCE), c) histogram of the PCE of 40 devices, and d) stable output *J*_max_ and PCE curves for the optimized pristine and GA‐ or TGA‐modified PSC devices.

To assess the effectiveness of GA modification and test it against the TGA treatment, we fabricated and optimized PSC devices based on the n‐i‐p planar architecture of glass/FTO/TiO_2_/perovskite/spiro‐OMeTAD/Au. In order to find the optimal concentration of the modifier, we prepared a series of perovskite precursors with different amounts of GA (i.e., 1, 2, 3, 5, and 10 mg mL^−1^) as well as TGA (e.g., 1, 5, 10, 15, and 20 µL mL^−1^). The corresponding device parameters are summarized in Table S3 (Supporting Information). When increasing the amount of GA from 0 to 5 mg mL^−1^, the PSC parameters including the short‐circuit current (*J*_sc_), fill factor (FF), open‐circuit voltage (*V*_oc_), and efficiency first increase and then decrease. The best device performance is found for 3 mg mL^−1^ GA incorporation. In contrast, when increasing the concentration of TGA from 1 to 20 µL mL^−1^ the PSC device performance gradually decreases, so there is no optimal concentration. This can be ascribed to the rough and pinhole rich surface of perovskite films. The champion and average photovoltaic performance parameters of pristine and GA‐ or TGA‐modified PSC devices are summarized in Table S4 (Supporting Information).The *J–V* curves of the champion solar cell devices based on GA‐and TGA‐modified perovskites were measured in reverse scan direction under an AM 1.5G solar simulator with an intensity of 100 mW cm^−2^ as shown in **Figure** **4**a (forward scans are displayed in Figure S12 in the Supporting Information). The highest PCE achieved for the control PSC device is 18.85% (*J*_sc_: 24.30 mA cm^−2^; *V*_oc_: 1.07 V; FF: 72.7%). Upon 3 mg mL^−1^ GA addition, a PCE of 21.32% (*J*_sc_: 25.13 mA cm^−2^; *V*_oc_: 1.08 V; FF: 78.2%) is achieved. Judging from the TRPL results, the enhanced *J*_sc_, *V*_oc_, and FF originate from the decreased trap density upon GA incorporation. The calculated *J*_sc_ values (24.04 mA cm^−2^ for the control PSC device, 24.25 mA cm^−2^ for the TGA‐modified PSC device, and 24.71 mA cm^−2^ for the GA‐modified PSC device) obtained from integrating over the external quantum efficiency (EQE) spectra shown in Figure 4b are in good agreement to the measured *J*_sc_ values. The PCE distribution for a batch of 40 individual perovskite solar cell devices is shown in Figure 4c with good reproducibility of the control, TGA‐, and GA‐modified PSC devices. The stabilized output curves of photocurrent density and PCEs as a function of time at the maximum powder condition for the control, TGA‐, and GA‐modified PSC devices are shown in Figure 4d. The GA‐modified PSC exhibits a stabilized PCE of 20.85% while the control and TGA‐modified PSC devices show lower values (18.12% and 16.01%, respectively) and a less stable behavior over time. So notably, the utilization of GA can also effectively enhance the PSC light stability. Moreover, the hysteresis effect of the PSCs with and without GA or TGA is investigated and the corresponding reverse (RS) and forward (FS) *J–V* scans are shown in Figure S12 (Supporting Information). The data are summarized in Table S5 (Supporting Information). Hysteresis indices of 13.3 for the control device, 16.6 for the TGA‐modified PSC, and 6.5 for the GA‐modified PSC are obtained. Therefore, the reduction in trap density by GA is also beneficial for lowering the hysteresis in PSC devices,^39^ while TGA has the opposite effect.

**Figure 5:**
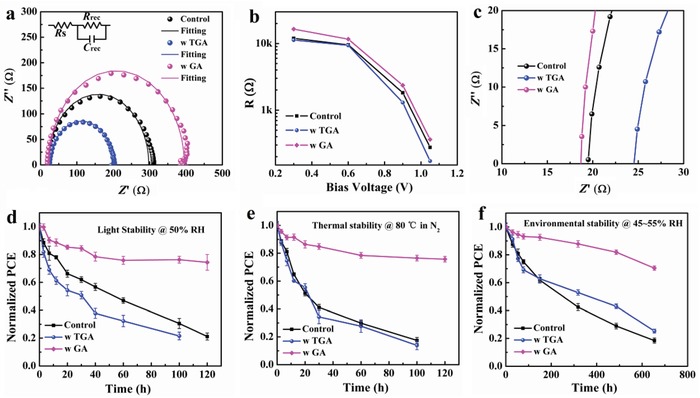
a) Nyquist plots of the PSC devices with and without GA or TGA at 1.05 V bias. (the inset shows the equivalent circuit model). b) Dependence of the fitted *R*_rec_ on different bias voltages (−0.3, −0.6, −0.9, and −1.05 V) for the PSC devices with and without GA or TGA. c) *R*_s_ values under 1.05 V bias for the PSC devices with and without GA or TGA. d) Light stability of PSC devices with and without GA or TGA, tested for 120 h under a xenon lamp at a relative humidity of ≈50%. e) Thermal stability of PSC devices with and without GA or TGA at 80 °C under N_2_ atmosphere. f) The dark environmental stability of the PSC devices with and without GA or TGA under a relative humidity of ≈55%.

**Figure** **5**a displays electric impedance spectroscopy (EIS) measurements of the fabricated PSC devices measured in dark at a bias of −1.05 V. The equivalent circuit model is shown in the inset and includes a series resistance (*R*_s_) and a recombination resistance (*R*_rec_) in parallel with a chemical capacitance (*C*_µ_) at the TiO_2_/perovskite/HTL interface.^54^, ^55^, ^56^, ^57^, ^58^ It can be seen that GA‐modified PSC device shows the largest *R*_rec_ (363 Ω) compared to the control device (277 Ω) and TGA‐modified PSC device (172 Ω). Additionally, the Nyquist plots of these PSC devices at different bias voltages (−0.3, −0.6, −0.9, and −1.05 V) were also carried out in the dark and the dependence of the fitted *R*_rec_ on these bias potentials is illustrated in Figure 5b. When increasing the bias voltage, the fitted *R*_rec_ values significantly decrease. For the same bias voltage, the *R*_rec_ value varies in the order of TGA < control < GA, which is in good agreement with the *V*_oc_ trend of the PSC devices as shown in Table S4 (Supporting Information). The series resistance of GA‐modified PSC device, as shown in Figure 5c, also shows a lower value (18.7 Ω) compared to the control (19.5 Ω) and TGA‐modified (24.5 Ω) PSC device, which demonstrates that GA can effectively reduce the series resistance of the PSC device and hence enhance the FF value.

In order to investigate the effects of perovskite modification on the device stability under external stress, we tested the effects of light soaking, thermal degradation, and moisture^59^, ^60^, ^61^ as shown in Figure 5d–f. The control PSC device only retains about 20% of its original PCE after 100 h illumination (at one sun, without UV filter, humidity level of 50%) and less than 20% after 100 h storage at 80 °C in nitrogen atmosphere. Without illumination or heat treatment it also drops to 20% of the initial value after 654 h storage under atmosphere with humidity of 45–55%. Under the same conditions, GA‐based PSC devices can maintain 74% of the original PCE after 120 h light soaking, 76% after 120 h thermal degradation, and 70% after 654 h moist test due to its superior film formation. Although TGA‐based PSC device shows 7% higher humid stability than the control device after 654 h, both perform significantly worse than the GA‐modified perovskite solar cells.

## 3 Conclusion

In this work, we presented the use of glycolic acid (GA) in the precursor solution of FA_0.85_MA_0.15_PbI_3_ perovskite. This material is solid at room temperature, but leaves the perovskite film upon annealing to 150 °C after DMSO sublimation, which is totally different from the common additive approaches. During processing, the molecule slows down the perovskite crystal growth process and simultaneously facilitates the generation of large grained and smooth perovskite films due to the strong interaction between GA and Pb^2+^. The improved film formation reduces the defect density in the perovskite and for the champion solar cell device a PCE as high as 21.32% is achieved. To understand the role of volatility, the less‐volatile molecule thioglycolic acid (TGA) with a similar chemical structure was investigated as an additive. This compound also increased the grain sizes in the perovskite film, however TGA remained in the film after annealing, leading to the formation of pinholes. Overall the perovskite surface was rougher and an increased defect density was found accompanied by a decline in PCE. This shows that the low evaporation point of GA is an essential feature. Furthermore, due to the superior film formation, GA can effectively enhance the light, thermal, and moisture stability of the PSC compared with a pristine or TGA‐modified film. In a word, we demonstrate a novel strategy for efficacious regulating perovskite crystallinity using volatile solid GA against nonvolatile TGA additive following DMSO sublimation in FA_0.85_MA_0.15_PbI_3_ PSCs, resulting in reduced defect density, enhanced device performance, and superior long‐term stability.

## Conflict of Interest

The authors declare no conflict of interest.

## Supporting information

Supporting Information
