# Supplementary material for: Solvent Engineering Using a Volatile Solid for Highly Efficient and Stable Perovskite Solar Cells
Source: Adv Sci (Weinh). 2020 Mar 10;7(10):1903250. doi: 10.1002/advs.201903250 (PMC7237837; doi:10.1002/advs.201903250)
Supplement: Supplementary file 1 — Supporting Information [file ADVS-7-1903250-s001.pdf]

Copyright WILEY-VCH Verlag GmbH & Co. KGaA, 69469 Weinheim, Germany, 2020.

ADVANCED SCIENCE
Open Access

Supporting Information

for *Adv. Sci.,* DOI: 10.1002/advs.201903250

Solvent Engineering Using a Volatile Solid for Highly Efficient and Stable Perovskite Solar Cells

Guohua Wu, Hua Li, Jian Cui, Yaohong Zhang, Selina Olthof, Shuai Chen, Zhike Liu, Dapeng Wang,* and Shengzhong (Frank) Liu**

Supporting Information

Solvent engineering using a volatile solid for high efficient and stable perovskite solar cells

Guohua Wu[a], *Hua Li*[a], *Jian Cui*[a], *Yaohong Zhang*[b]*, *Selina Olthof*[ac], *Shuai Chen*[a], *Zhike Liu*[a], *Dapeng Wang*[a]*, *Shengzhong (Frank) Liu*[a]*

[a] Key Laboratory of Applied Surface and Colloid Chemistry, National Ministry of Education, Shaanxi Key Laboratory for Advanced Energy Devices, Shaanxi Engineering Laboratory for Advanced Energy Technology, School of Materials Science and Engineering, Shaanxi Normal University, Xi'an 710119, China.

[b] Faculty of Informatics and Engineering, The University of Electro-Communications, 1-5-1 Chofugaoka, Chofu, Tokyo 182-8585, Japan.

[c] Department of Chemistry, University of Cologne, Luxemburger Street 116, 50939 Cologne, Germany.

Experimental

1. Experimental Section

1.1. Materials

Dimethyl-formamide (DMF, 99%), dimethyl sulfoxide (DMSO, 99.9%), and chlorobenzol (CB, 99.8%) were purchased from Sigma-Aldrich. Lead iodide (PbI_2, 99%), spiro-OMeTAD, MAI, and FAI were purchased from Xi'an Polymer Light Technology Corporation. GA and TGA were purchased from J&K Scientific Ltd.

1.2. Synthesis of adduct powders

For PbI_2•DMSO adduct, 1mmol PbI_2 are dissolved in 1mmol DMSO. Then, 10 mL of toluene is added to precipitate the PbI_2•DMSO adduct. For PbI_2•DMSO•GA and PbI_2•DMSO•TGA adducts, 1 mmol PbI_2 and GA or TGA are dissolved in 1 mmol DMSO. Then, 10 mL of toluene is added to precipitate the PbI_2•DMSO•GA and PbI_2•DMSO•TGA adducts. For FAI•PbI_2•DMSO, 10 mL toluene is added into a 600 mg DMF solution of 1 mmol PbI_2, FAI, and DMSO to precipitate the corresponding adduct. For FAI•PbI_2•DMSO•GA and FAI•PbI_2•DMSO•TGA adducts, 10 mL toluene is added into a 600 mg DMF solution of 1 mmol PbI_2, FAI, DMSO, and GA or TGA to precipitate the corresponding adducts. All the precipitates are used after drying under vacuum under 50 ^{o}C.

1.3. Device fabrication.

Fluorine-doped tinoxide (FTO) glass substrate were cleaned using ethanol and then immersed in a 40 mM $TiCl_4$ aqueous solution at 70 °C for 1 h. Next, the FTO/TiO_2 substrates were annealed at 200 °C for 30 min in air. Afterwards the perovskite

precursor solution was spin-coated on the FTO/TiO_2 substrate followed by an antisolvent drip of chlorobenzene (CB) solution and subsequently annealed at 150 °C for 30 min to form the perovskite film. The spiro-OMeTAD layer was spin-coated onto the perovskite films. Finally, an 80-nm-thick gold electrode was thermally evaporated onto the stack.

Here, the pristine perovskite precursor solution was prepared by dissolving FAI, MAI, and PbI_2 in 1 ml DMF/DMSO (v/v 4:1) with a molar ratio of 0.85:0.15:1. 3 mg/ml GA or 1μl /ml TGA was added into the above solution to prepare the GA or TGA modified perovskite films and PSC devices. The chemicals including glycolic acid (GA) and thioglycolic acid (TGA) are purchased from J&K Scientific company without further purification.

1.4. Instruments and Characterization.

Surface morphology or cross-section images of the perovskite films or PSC devices were obtained using a field emission scanning electron microscope (Jeol SU-8020) equipped with energy dispersive X-ray spectroscopy (EDX). The water contact angle measurements for the perovskite films were carried out on an OCA20 instrument. Atomic Force Microscope (AFM) images of the perovskite films were captured by using a NanoScope IV SPM Control Station with a silicon cantilever. X-ray diffraction (XRD) patterns were acquired using a Bruker D8 GADDS diffractometer with the Cu Kα radiation. The absorption spectra of the annealed perovskite films were obtained by a Shimadzu UV-3600 spectrophotometer. An Edinburgh Instruments Ltd. FLS980 spectrometer was used to measure the steady state and

transient state photoluminescence (PL) spectra. The density functional theory (DFT) calculation were implemented in VASP (Vienna *ab*-initio simulation package) using the Perdew-Burke-Ernzerhof (PBE) function and Tkatchenko-Scheffler (TS) method was applied to conduct the calculation of the van der Waals interactions. The Fourier Transform infrared spectroscopy (FTIR) of GA, TGA, and their adduct compounds was performed on a Bruker EQUINX55 spectrometer. The thermogravimetric analysis of the adduct compounds was conducted under N_2 atmosphere from room temperature to 700 ^oC with a 20 ^oC/min rate. The current density−voltage ($J-V$) curve of the PSC was obtained by using a Keithley Model 2400 digital source meter under an illumination of an AM 1.5 solar simulator (100 $mWcm^{-2}$, SAN-EI, Enlitech). All PSC devices were measured with a 20 mV step voltage and a 10 ms delay. The scan rate is 100 mV S^{-1}. The external quantum efficiency (EQE) spectra of the PSCs were measured using a QTest Station 500TI monochromator. EIS measurements were conducted on a Zahner Zennium electrochemical workstation under dark condition with a frequency range from 4×10^6 to 10 Hz and a 50 mV amplitude.

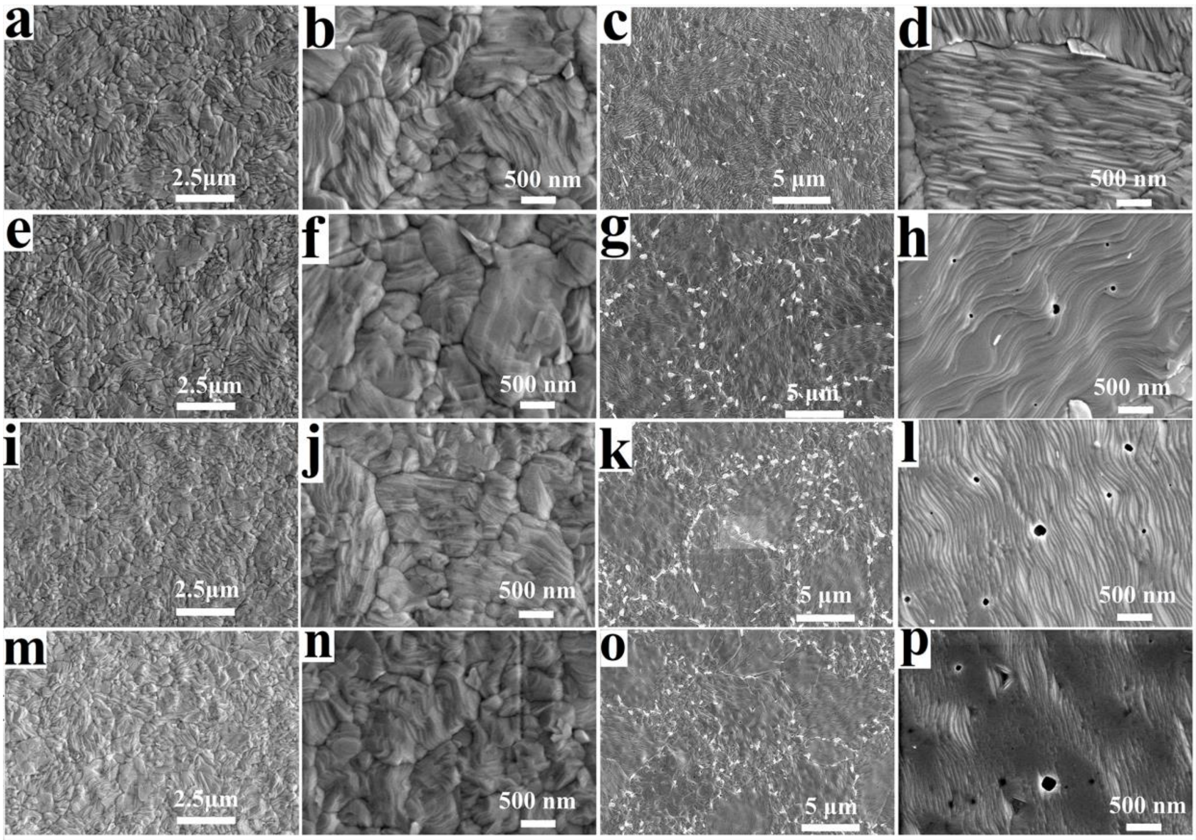

Figure S1. (a) High-resolution scanning electron microscopy (SEM) images of 1 mg/ml (a, b), 2 mg/ml (e, f), 5 mg/ml (i, j), or 10 mg/ml (m, n) GA and 5 μl/ml (c, d), 10 μl/ml (g, h), 15 μl/ml (k, l), or 20 μl/ml (o, p) TGA modified perovskite films.

Table S1. Summary of fitting parameters of time-resolved PL spectra for the perovskite films with or without GA or TGA.

Sample	τ_1 (ns)	Amplitude τ_1 (%)	τ_2 (ns)	Amplitude τ_2 (%)	τ_{ave} (ns)
control	64.40	87.88	25.43	12.12	59.68
GA	102.85	92.07	28.61	7.93	96.96
TGA	48.64	59.33	16.35	40.67	35.51

Table S2. Calculated interaction energies of PbI_2•DMSO, PbI_2•GA, PbI_2•TGA, FA^+•DMSO, FA^+• GA, FA^+•TGA, FAI•PbI_2•DMSO, FAI•PbI_2•GA, and FAI•PbI_2•TGA using DFT calculation.

**Molecules**	**Binding energy (eV)**
PbI_2•DMSO	-0.840
PbI_2•GA	-0.955 (PbI_2•GA-1) /-0.613 (PbI_2•GA-2)
PbI_2•TGA	-0.989 (PbI_2•TGA-1) /-0.817 (PbI_2•TGA-2)
FA^+•DMSO	-1.347
FA^+•GA	-1.286 (FA^+•GA-1) /-1.286 (FA^+•GA-2)
FA^+•TGA	-1.214 (FA^+•TGA-1) /-1.254 (FA^+•TGA-2)
FAI•PbI_2•DMSO	-1.877
FAI•PbI_2•GA	-2.356 (FAI•PbI_2•GA-1) /-2.292 (FAI•PbI_2•GA-2)
FAI•PbI_2•TGA	-2.496 (FAI•PbI_2•TGA-1) /-1.842 (FAI•PbI_2•TGA-2)

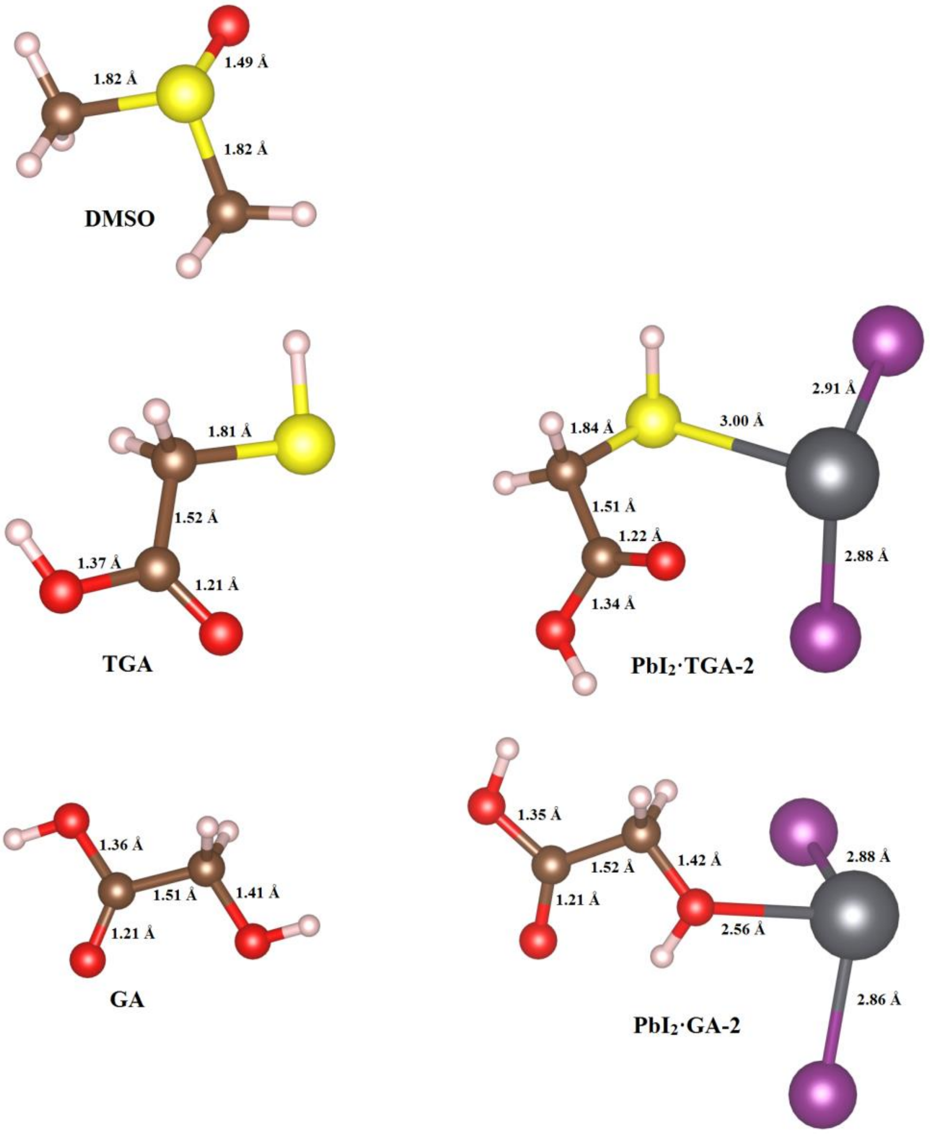

Figure S2. The optimized molecular structures of DMSO, TGA, GA, PbI_2•DMSO, PbI_2•GA-2, and PbI_2•TGA-2.

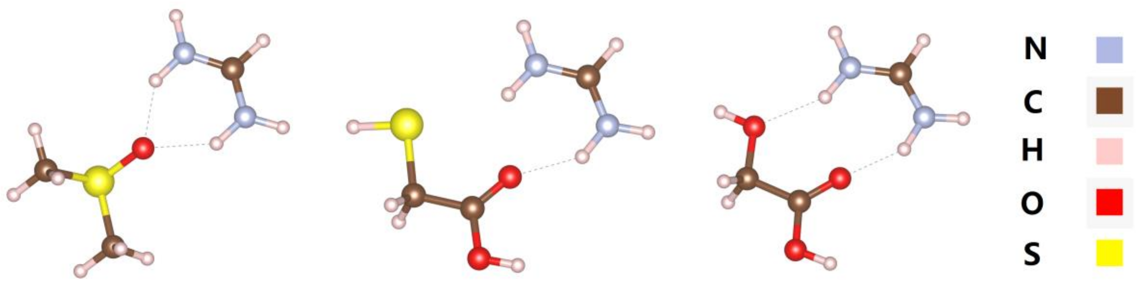

FA$^+$•DMSO **FA$^+$•TGA** **FA$^+$•GA**

Figure S3. The optimized molecular structures of FA$^+$•DMSO, FA$^+$•TGA, and FA$^+$•GA with the largest binding energy.

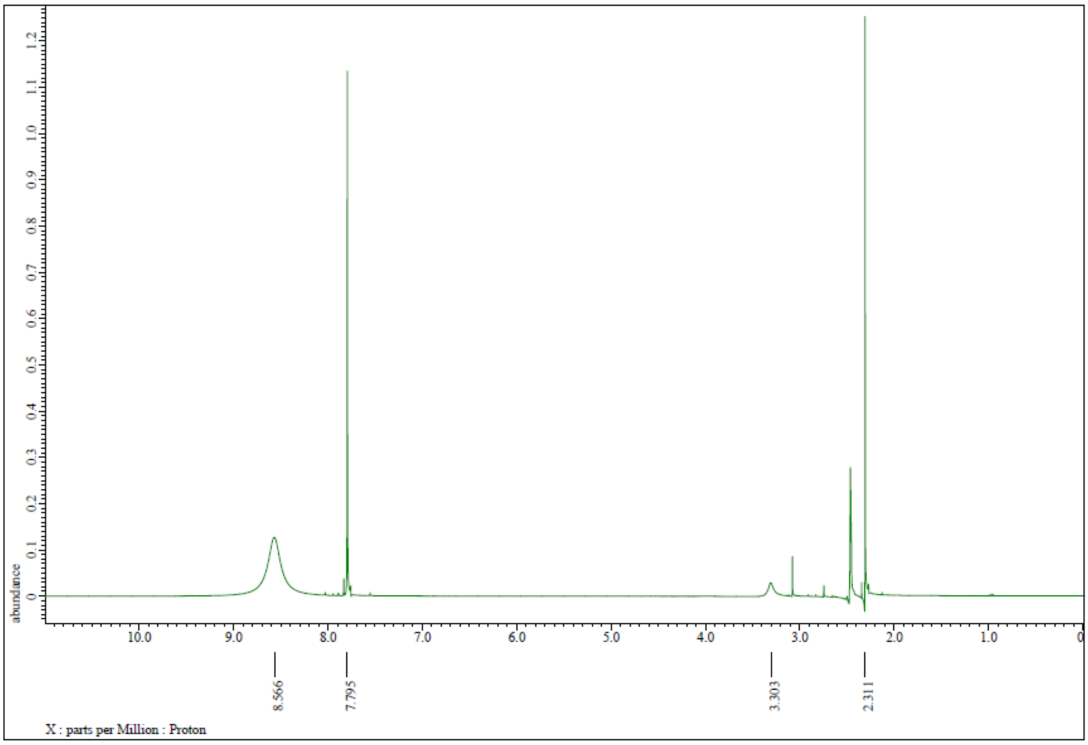

Figure S4. The ^{1}H NMR of $FA_{0.85}MA_{0.15}PbI_3$ perovskite precursor solution.

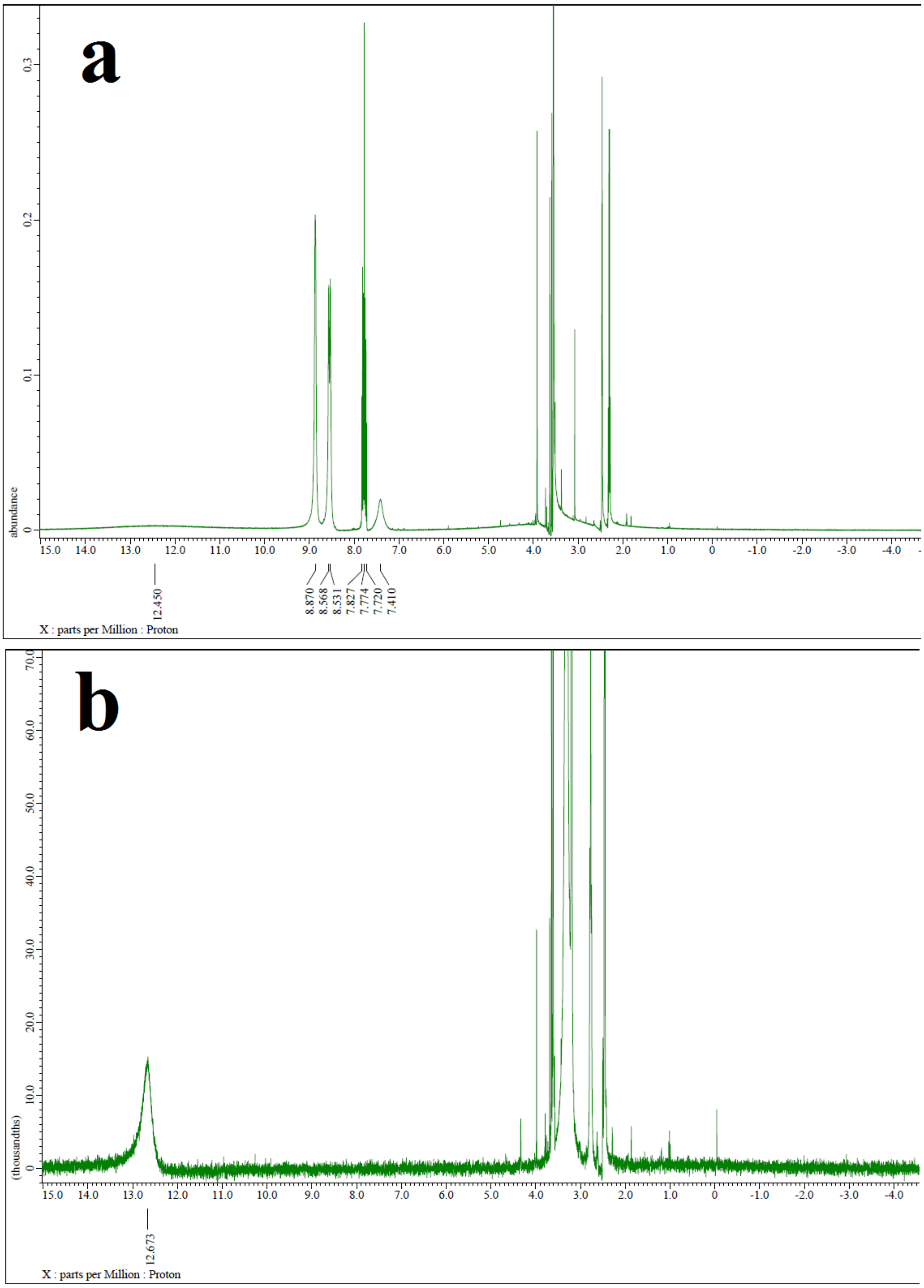

Figure S5. The ^{1}H NMR of $FA_{0.85}MA_{0.15}PbI_3$ perovskite precursor solution with TGA (a) and TGA in d_6-DMSO (b).

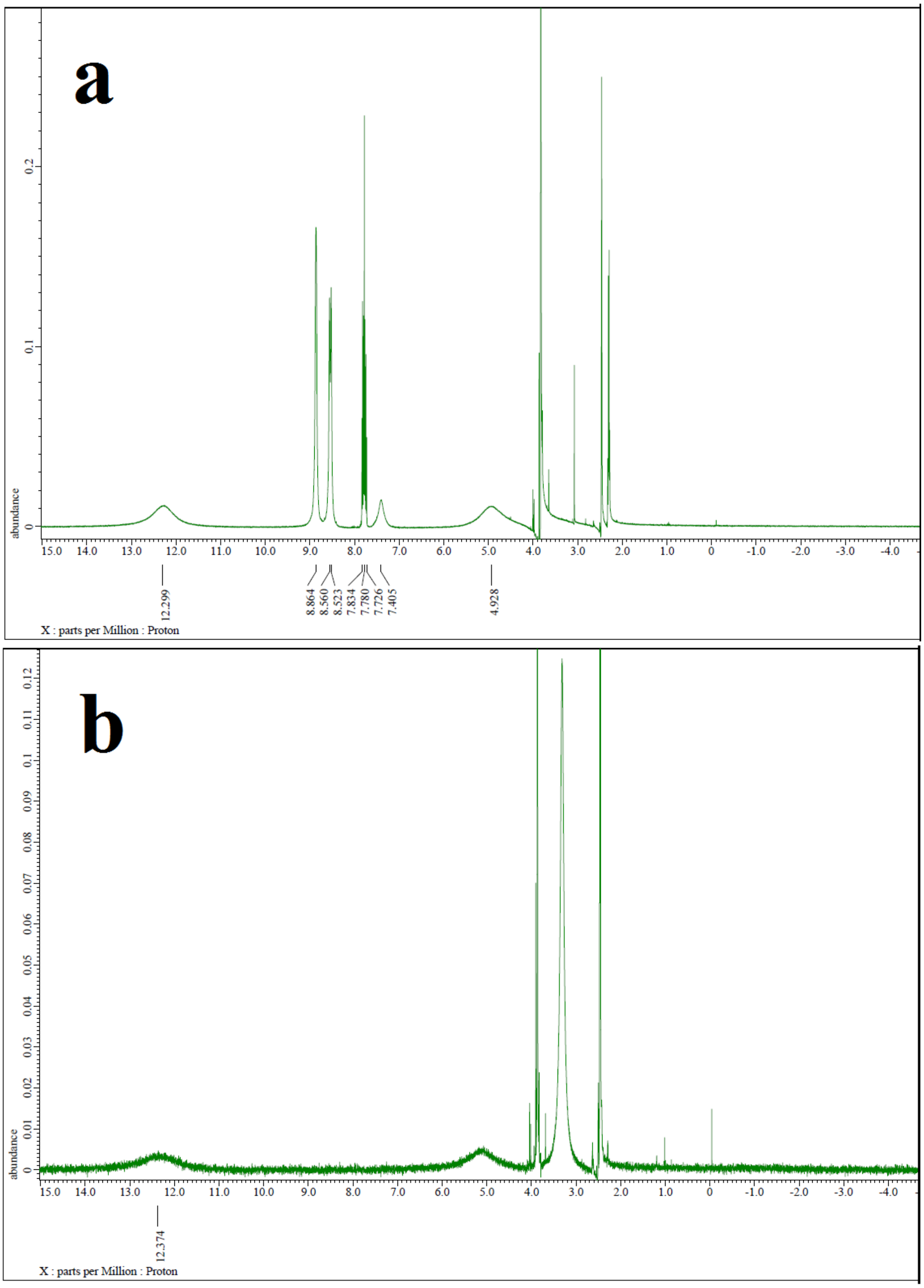

Figure S6. The ^{1}H NMR of $FA_{0.85}MA_{0.15}PbI_3$ perovskite precursor solution with GA (a) and GA in d_6-DMSO (b).

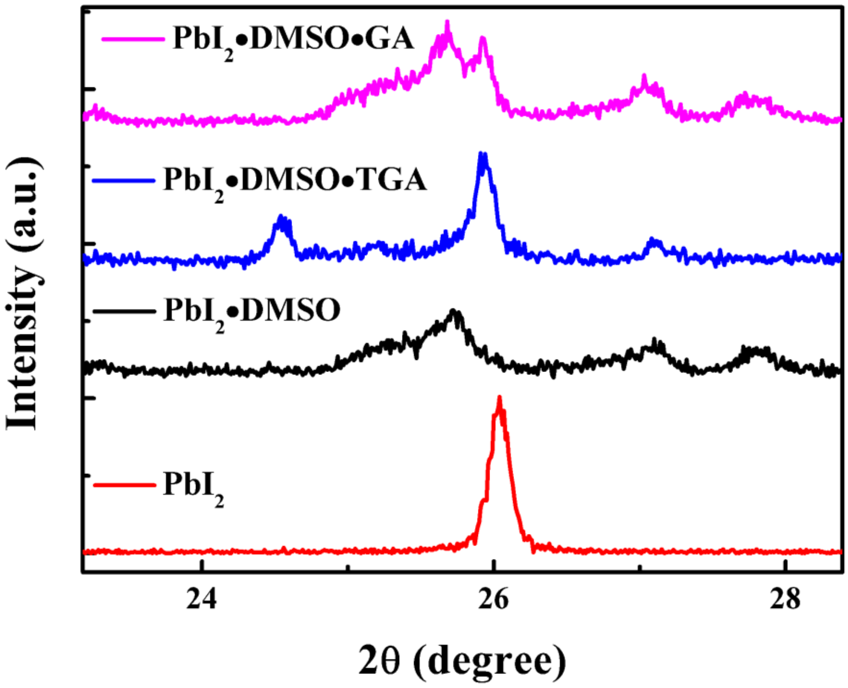

Figure S7. XRD patterns of the as-formed PbI_2 adducts compared with PbI_2 with a 2 θ range from 23 degree to 28 degree.

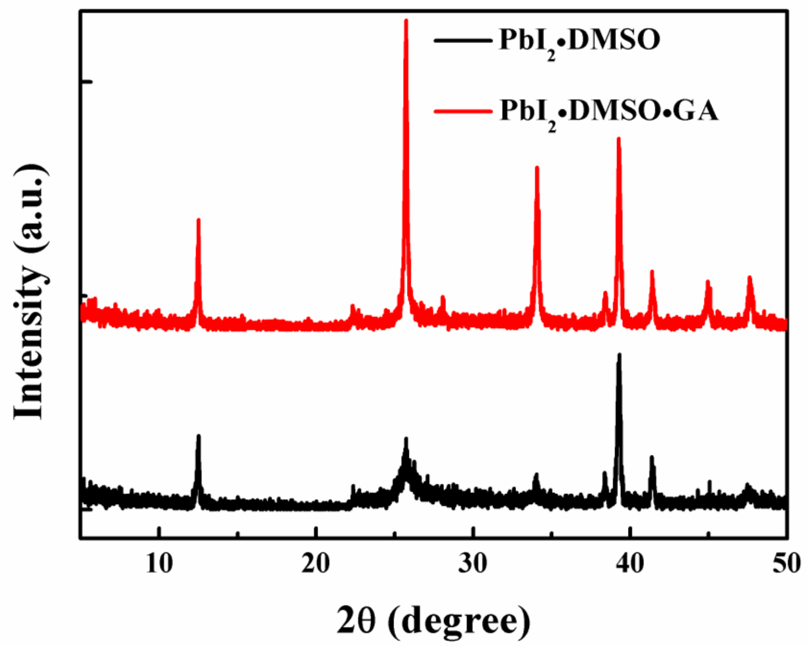

Figure S8. XRD patterns of PbI_2•DMSO and PbI_2•DMSO•GA after a temperature of 150 ^{o}C with a 2 θ range from 5 degree to 50 degree.

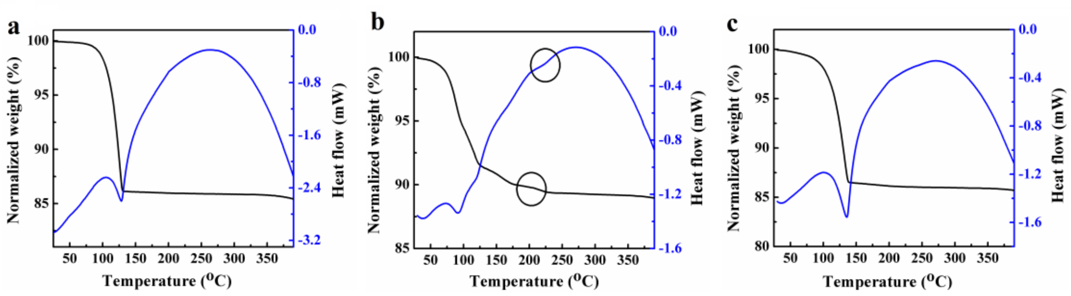

Figure S9. Thermogravimetric analysis (TG) of PbI_2•DMSO (a), FAI•PbI_2•DMSO•TGA (b), and FAI•PbI_2•DMSO•GA (c) adducts.

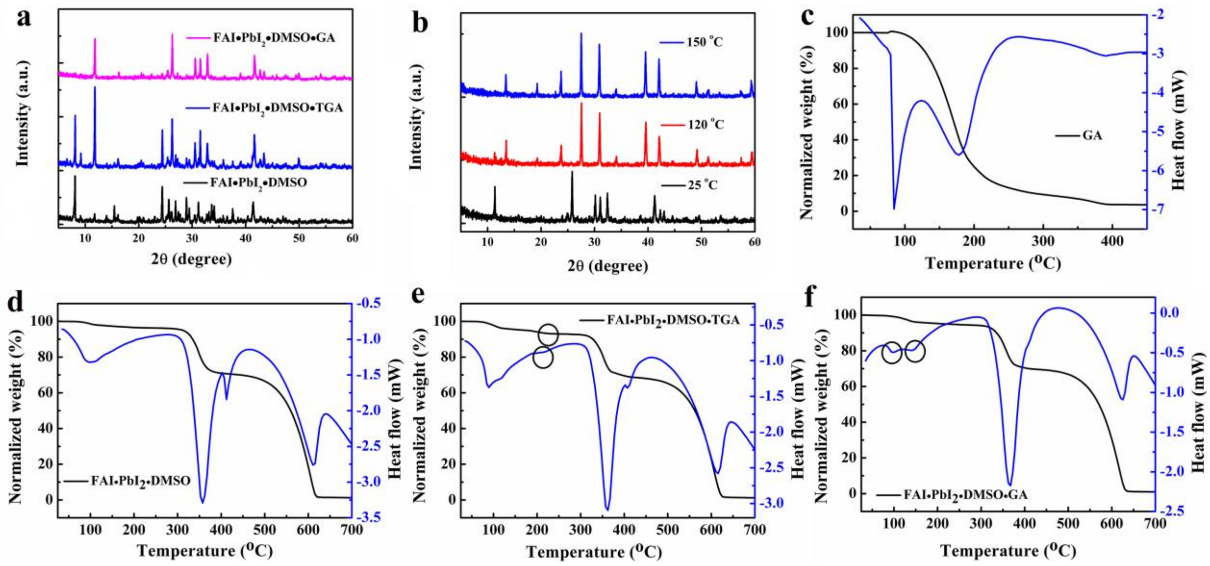

Figure S10. (a) XRD patterns for as-formed FAI•PbI_2•DMSO, FAI•PbI_2•DMSO•TGA, and FAI•PbI_2•DMSO•GA adducts, (b) XRD patterns for annealed FAI•PbI_2•DMSO•GA adduct, thermogravimetric analysis (TG) of GA (c), FAI•PbI_2•DMSO (d), FAI•PbI_2•DMSO•TGA (e), and FAI•PbI_2•DMSO•GA (f) adducts.

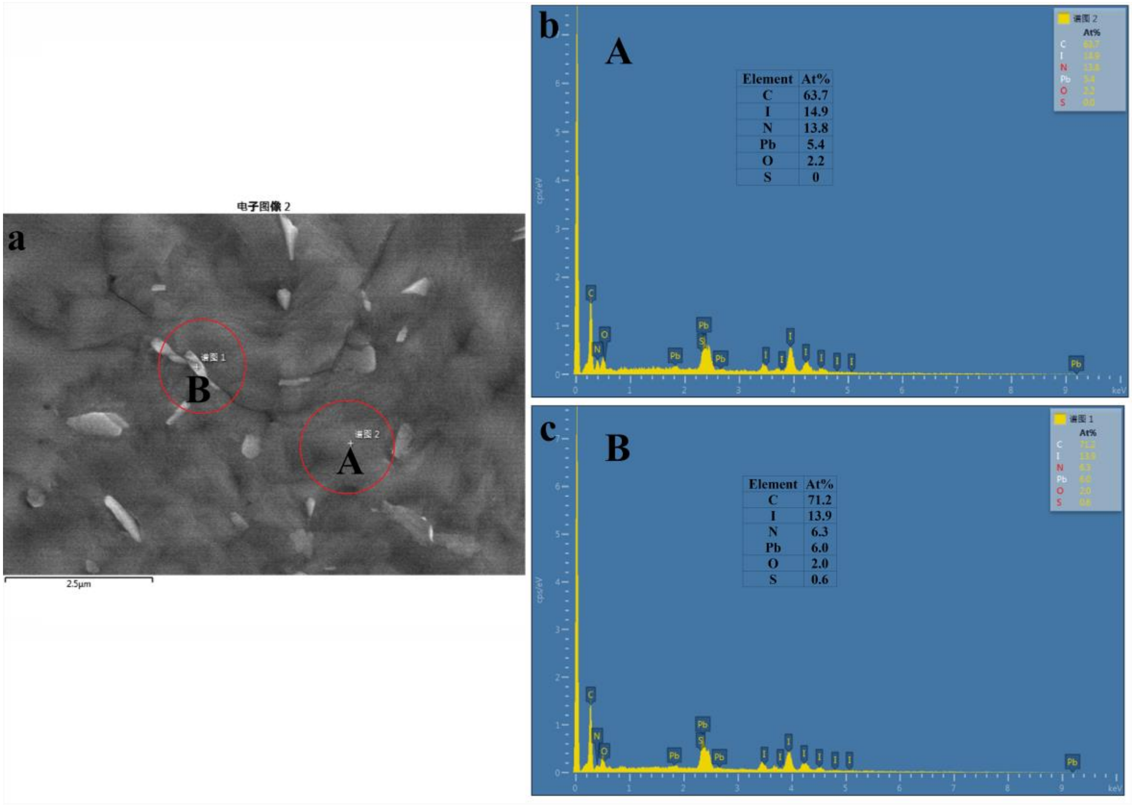

Figure S11. (a) SEM image of perovskite film containing the TGA additive. (b) and (c) are the EDX spectra of point A and B in (a), revealing the presence of TGA (based on sulfur peak) in B.

Table S3. Summary of the best photovoltaic parameters of the PSCs with different amounts of TGA or GA using a reverse scan direction.

Samples	amount	V_{oc} (V)	J_{sc} (mA/cm^2)	FF (%)	PCE (%)
Control	-	1.07	24.30	72.7	18.85
TGA	1 µl/ml	1.02	24.99	70.0	17.79
	5 µl/ml	1.00	24.84	70.3	17.54
	10 µl/ml	0.99	24.70	69.3	16.92
	15 µl/ml	0.97	24.67	67.9	16.24
	20 µl/ml	0.94	24.97	68.3	16.03
GA	1 mg/ml	1.07	24.48	77.1	20.26
	2 mg/ml	1.07	24.67	79.3	20.91
	3 mg/ml	1.08	25.13	78.2	21.32
	5 mg/ml	1.07	24.71	77.4	20.46
	10 mg/ml	1.06	24.67	77.9	20.41

Table S4. Photovoltaic performance parameters of pristine and GA or TGA modified PSC devices.

PSCs		V_{oc} (V)	J_{sc} (mA/cm^2)	FF (%)	PCE (%)
Control	Best	1.07	24.30	72.7	18.85
	Average	1.05±0.02	24.33±0.27	72.4±1.2	18.50±0.37
1μl/ml TGA	Best	1.02	24.99	70.0	17.79
	Average	0.98±0.03	24.71±0.28	67.4±2.6	16.30±1.49
3mg/ml GA	Best	1.08	25.13	78.2	21.32
	Average	1.07±0.01	24.58±0.55	78.4±1.0	20.62±0.70

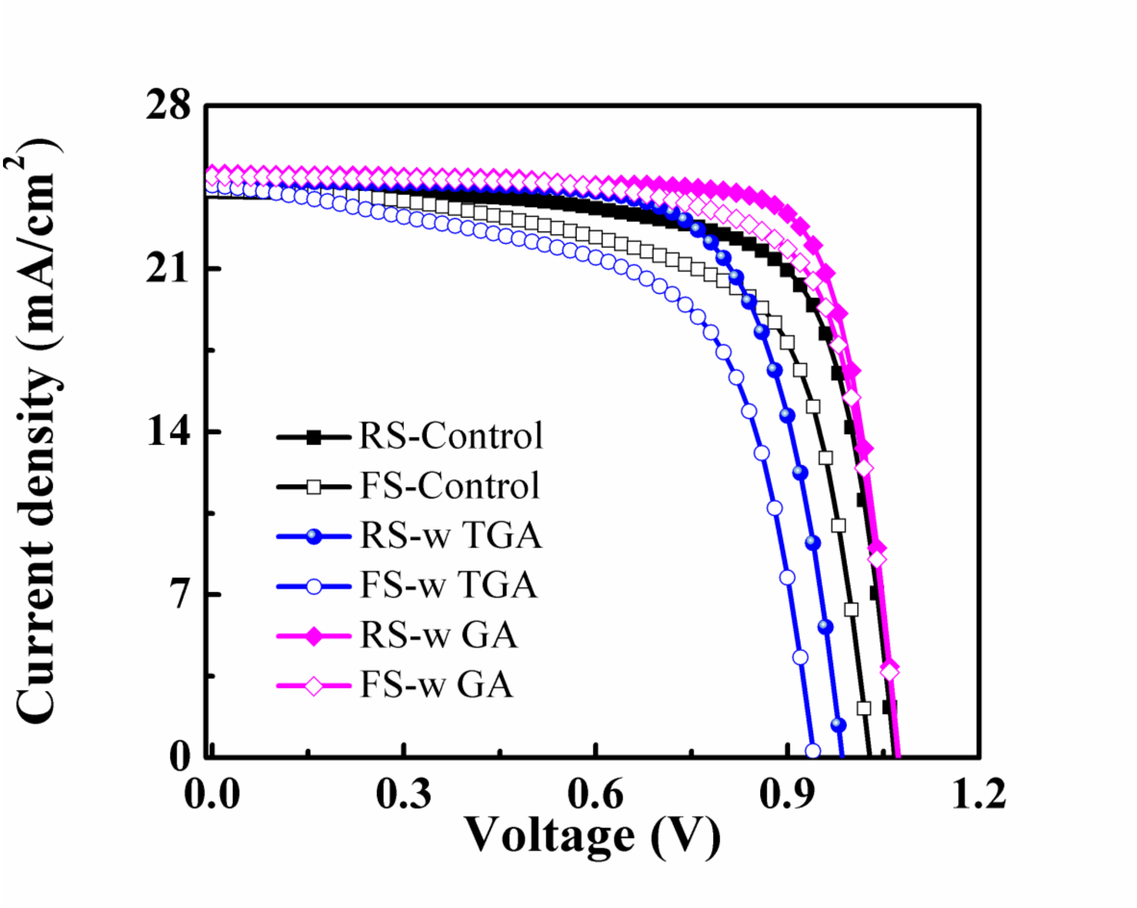

Figure S12. *J*-*V* curves of PSCs with and without GA or TGA measured by forward (short circuit-to-open circuit) and reverse (open circuit-to-short circuit) scans with 0.02 V voltage step.

Table S5. Summary of photovoltaic parameters of the PSCs with and without TGA or GA under reverse and forward voltage scans.

PSCs	Scan direction	V_{oc} (V)	J_{sc} (mA/cm^2)	FF (%)	PCE (%)	Hysteresis Index (%)
Control	Reverse	1.07	24.30	72.7	18.85	13.3
	Forward	1.03	24.39	66.3	16.63	
TGA	Reverse	0.99	24.78	70.7	17.26	16.6
	Forward	0.94	24.59	62.2	14.40	
GA	Reverse	1.07	25.07	78.1	21.03	6.5
	Forward	1.07	24.97	73.4	19.67	
